# Serum Homocysteine Level Is Positively Correlated With Serum Uric Acid Level in U.S. Adolescents: A Cross Sectional Study

**DOI:** 10.3389/fnut.2022.818836

**Published:** 2022-03-29

**Authors:** Yumeng Shi, Zuxiang Wu, Ji Wu, Zhiqiang Chen, Ping Li

**Affiliations:** Department of Cardiovascular Medicine, The Second Affiliated Hospital of Nanchang University, Nanchang, China

**Keywords:** serum homocysteine levels, serum uric acid, adolescents, NHANES, cross-sectional study

## Abstract

**Background:**

Physiologically, the levels of homocysteine (Hcy) and serum uric acid (SUA) are closely related; however, clinical studies on the relationship between Hcy and SUA have drawn different conclusions and have not analyzed this association among adolescents. This study therefore aimed to evaluate the relationship between Hcy and SUA levels among adolescents.

**Methods:**

In this study, we performed a cross-sectional analysis of data from the National Health and Nutrition Examination Survey for the period 1999–2006, which included 5,404 adolescents aged 12–19 years. An elevated SUA level was defined as ≥5.5 mg/dL. Multivariate logistic regression and multivariate linear regression models were also applied in this study.

**Results:**

The mean concentrations of Hcy and SUA were 6.0 μmol/L and 5.0 mg/dL, respectively, and 33.6% of the participants had SUA levels of ≥5.5 mg/dL. There was a dose–response relationship between Hcy and SUA, and Hcy was linearly positively correlated with SUA. The β value [95% confidence interval (CI)] for SUA in the fully adjusted model was1.43 (95% CI: 1.18, 1.68). The multivariate logistic regression model showed that per 1 increment in log-transformed Hcy, the risk of elevated SUA levels increased by 8.80 times (odds ratio, 8.80, 95% CI: 4.25, 18.20). Subgroup analyses showed that the relationship between Hcy and SUA was significantly different according to sex, age, body mass index (BMI), and estimated glomerular filtration rate (eGFR) stratification (P for interaction <0.05).

**Conclusion:**

Hcy levels were positively correlated with SUA levels and elevated SUA levels among U.S. teenagers, and this effect was more significant among boys aged ≥17 years and among people with lower BMI and eGFR.

## Background

Homocysteine (Hcy) is a sulfur-containing amino acid that is not obtained from the diet but is mainly synthesized in the methionine (MeT) cycle (1). An increase in Hcy levels is considered a risk factor for cardiovascular diseases (CVD) (2). Hcy causes damage to vascular endothelial function and eventually leads to CVD (3). Although it is usually easier to observe the clinical manifestations of CVDs in adulthood, there is evidence that these diseases may begin in childhood and adolescence (4). An observational study showed that more than 50% of children with hereditary homocysteinemia died due to premature vascular diseases and concluded that Hcy is a causal risk factor for CVDs in children (5). Previous studies have also shown that supplementation of B vitamins such as folic acid can reduce Hcy level and prevent the occurrence and development of cardiovascular diseases (6–9). It is essential to identify early changeable risk factors for CVD among adolescents to prevent the occurrence and development of CVD in adulthood.

Serum uric acid (SUA), like Hcy, is a risk factor for CVD. Most studies have shown that an increase in the SUA level plays a vital role in CVD occurrence (10–12). SUA causes endothelial dysfunction, thus increasing oxidative stress and causing microvascular diseases, which can induce the proliferation of vascular smooth muscle cells and reduce the bioavailability of endothelial nitric oxide (13). According to relevant studies, an elevated SUA level is defined as ≥5.5 mg/dL (14, 15). The potential mechanism underlying the relationship between uric acid and Hcy is as follows: the MeT cycle occurs in the human body; that is, MeT can be converted into S-adenosylhomocysteine (SAH), which can then be converted into Hcy and adenosine (16), Hcy receives methyl MeT regeneration (17), and adenosine can be metabolized into uric acid (18). Hcy and uric acid levels could be positively correlated according to the above physiological mechanisms. However, previous studies on the associations between Hcy and SUA are scarce, and most of them were carried out among healthy adults, patients with arteriosclerosis vascular disease (ASCVD), diabetes patients, and gout patients (19–23). Moreover, the above studies have reported inconsistent results on the association between Hcy and SUA.

To explore the above problems, this study used data from the National Health and Nutrition Examination Survey (NHANES) for the period 1999–2006 to evaluate the relationship between Hcy and SUA among US adolescents.

## Materials and Methods

### Study Population and Design

The NHANES is a population-based cross-sectional survey that collects information on the health and nutrition of American families. The project includes two parts: an in-home interview and physical examination. The survey was conducted at the participants’ homes. The NHANES agreement was approved by the Review Committee of the National Center for Health Statistics Research Ethics. All adult participants provided written informed consent, and those under 18 years of age were required to submit the consent of their parents or guardians. The NHANES adopts a stratified multistage sampling design to obtain representative samples of American residents (24, 25). More detailed information can be obtained from https://www.cdc.gov/nchs/nhanes/index.htm. The NHANES dataset is available at DataDryad https://doi.org/10.5061/dryad.d5h62.

The data for this study were obtained from the NHANES database for the period 1999–2006. Fasting blood samples were collected from participants aged 12–19 years, including 8,374 teenagers. We excluded participants with missing Hcy values (*n* = 2,706), SUA values (*n* = 84), and dietary vitamin B12 intake (*n* = 180). Finally, 5,404 people were included in the final analysis (Supplementary Figure 1).

### Data Collection

A questionnaire survey, anthropometric measurements, and fasting blood sample collection were conducted by professionally trained researchers in participants’ homes following a standardized protocol. The questionnaire included questions on demographic characteristics such as sex, age, race, educational attainment (less than high school, high school, and high school or above), and dietary nutrition intake (vitamin B12 and vitamin B6 intake). Race/ethnicity included non-Hispanic whites, non-Hispanic blacks, Mexican Americans, other Hispanics, and other races. Anthropometric indicators included height, weight, and blood pressure (BP). Body mass index (BMI) was calculated as weight divided by height squared (kg/m2). After 8 h of fasting, venous blood samples including fasting blood glucose (FBG), total cholesterol (TC), triglyceride, serum creatinine, blood uric acid (SUA), blood urea nitrogen (BUN), C-reactive protein (CRP), serum vitamin B12, serum folic acid, aspartate aminotransferase (AST), alanine aminotransferase (ALT), and gamma-glutamyl transferase (GGT) were collected. A Zeeman background-corrected multi-element atomic absorption spectrometer was used to measure the blood levels. The CRP level was measured by latex-enhanced nephelometry on a Dade Behring Nephelometer II Analyzer System (BNII). Using the Jaffe kinetic alkaline picrate method, serum creatinine levels were measured using a Roche Hitachi 917 or 704 multichannel analyzer in 2001 and Beckman Synchron LX20 in 2002. According to the advice of the National Health and Nutrition Examination Survey, the serum creatinine level was calibrated and standardized with a gold standard method. The formula for estimated glomerular filtration rate (eGFR) is different in different groups of people, and the Schwartz formula is used to calculate eGFR in adolescents. Males: eGFR = 0.7 × (height in cm)/(serum creatinine in mg/dL); Females: eGFR = 0.55 × (height in cm)/(serum creatinine in mg/dL) (26, 27).

### Exposure Variable and Outcomes

The exposure variable in this study was Hcy, and two measurement methods were used to detect Hcy levels. In 2001, Abbott Homocysteine IMX (Hcy) Assay (Abbott Diagnostics, Abbott Park, IL, United States) was used, while during 2002–2006, Abbott Axsym System (Abbott Diagnostics, Abbott Park, IL, United States) was used. The long-term coefficient of variation of the NHANES from 2001 to 2006 was 3–5% of the total Hcy concentration. A cross-study of the two methods showed no significant difference between the two methods. Details of these testing and quality standards can be found at https://cdc.gov/nchs/nhanes.

The outcome variables were SUA and elevated SUA levels. SUA was measured using a Roche Hitachi 917 or 704 multichannel analyzer in 2001 and Beckman Synchron LX20 in 2002 using the colorimetric method. The distribution of uric acid results in laboratories in different periods was compared, and no significant difference was observed. Although there is no standard definition of hyperuricemia among adolescents, previous research reports show that an SUA level of ≥5.5 mg/dL was related to the risk of hypertension (14, 15). Therefore, in this study, we defined an SUA level of ≥5.5 mg/dL as elevated SUA.

### Statistical Analysis

The analysis was conducted according to the Centers for Disease Control and Prevention.^1^ Because the distribution of Hcy levels is skewed, the log-transformed Hcy (LgHcy) analysis of Hcy was carried out in our study. The data are expressed as mean ± SD or proportions. We used the suggested weighting method in the data analysis, considering the significant differences. Multivariate linear regression analysis and multivariate logistic regression analysis were used to evaluate the correlation among LgHcy, SUA, and elevated SUA levels. In addition, we ensured the robustness of the data analysis. We converted Hcy into tertiles and calculated the *P*-value. Regression analysis established three models: Model 1 was adjusted for age, sex, BMI, SBP, and DBP. Model 2 was adjusted for all covariables in Model 1 plus adjusted for non-Hispanic white, non-Hispanic black, Mexican American, other Hispanic, other races, and education. Model 3 was adjusted for all covariates in Model 2 plus adjusted for FBG, triglycerides, TC, eGFR, BUN, CRP, serum folate, serum vitamin B12, ALT, AST, GGT, vitamin B12 intake, and vitamin B6 intake. To test for the significant associations, the generalized additive model and fitting smooth curve (penalty spline method) were used to further explore the shape of their dose–response relationship. In subgroup analysis using hierarchical logistic regression analysis, possible modifications of the association between LgHcy and SUA were also evaluated for variables including sex (male vs. female), age (<17 vs. ≥17 years), BMI (<20.5 vs. 20.5–24.5 vs. ≥25 kg/m2), education attainment (less than high school vs. high school or higher), serum vitamin B12 (<452 vs. 452–623 vs. ≥623 pg/mL), serum folate (<10.1 vs. 10.1–14.3 vs. ≥14.3 ng/mL), and eGFR (<138 vs. 138–171 vs. ≥171 mL/min/1.73 m2).

All analyzed data were analyzed using the statistical software packages R^2^ and Empower (R) (Boston X & Y Solutions, Boston, MA, United States).^3^ Differences were considered statistically significant at *p* < 0.05.

## Results

### Baseline Characteristics

Based on the inclusion and exclusion criteria, 5,404 teenagers were included in this analysis, and the average age of participants in this study was 14.98 ± 2.01 years. Among these participants, 50.48% were boys, 25.44% were non-Hispanic white, 31.25% were non-Hispanic black, 35.47% were Mexican American, 4.13% were other Hispanic, and 3.70% were other races. The mean (SD) concentrations of Hcy and SUA were 6.0 (2.6) μmol/L and 5.0 (1.3) mg/dL, respectively, and 33.6% of the participants had SUA ≥ 5.5 mg/dL. The clinical characteristics of the study subjects are presented in Table 1. We found no significant difference in other Hispanic, other races, vitamin B12 intake, vitamin B6 intake, FBG, TC, triglyceride, and CRP in different Hcy groups. Compared with the other two groups, the participants in the group with higher Hcy were primarily males and older, with a higher proportion of non-Hispanic whites and blacks; higher education level; and higher levels of SUA, BUN, serum vitamin B12, AST, ALT, and GGT, but lower levels of SBP, DBP, eGFR, and serum folate (all *P* < 0.05).

**TABLE 1:** Weighted characteristics of study population based on LgHcy tertiles.

	LgHcy tertiles^†^, μ mol/L			
Characteristics*	T1 (<5.07)	T2 (5.07–6.34)	T3 (≥6.34)	*P-*value
N	1,793	1,799	1,812	
Age, year	14.17 ± 1.91	14.87 ± 1.95	15.88 ± 1.77	<0.001
Male, %	37.31%	49.14%	64.85%	<0.001
BMI, kg/m^2§^	22.86 ± 5.57	23.62 ± 5.85	24.38 ± 5.76	<0.001
SBP, mm Hg	107 ± 10	109 ± 10	111 ± 10	<0.001
DBP, mm Hg	60 ± 12	61 ± 11	61 ± 12	0.085
**Race**				
Non-Hispanic White,%	22.87%	24.51%	28.92%	<0.001
Non-Hispanic Black,%	28.61%	31.52%	33.61%	0.005
Mexican American,%	41.38%	36.41%	28.70%	<0.001
Other Hispanic,%	3.68%	4.00%	4.69%	0.297
Other races,%	3.46%	3.56%	4.08%	0.564
Education,%				<0.001
<High school	94.97%	93.27%	86.98%	
High school	2.62%	3.73%	7.56%	
>High school	2.40%	3.00%	5.46%	
**Dietary**				
Vitamin B12, μg/day	4.80 ± 4.13	5.00 ± 4.11	4.82 ± 4.87	0.300
Vitamin B6, μg/day	1.75 ± 1.18	1.79 ± 1.16	1.80 ± 1.41	0.421
Laboratory data				
FBG, mg/dL	86.82 ± 20.59	86.20 ± 12.50	86.39 ± 12.21	0.469
TC, mg/dL	162.35 ± 33.04	162.02 ± 29.48	161.55 ± 31.31	0.745
Triglycerides, mg/dL	86.75 ± 66.12	83.44 ± 52.37	84.42 ± 55.26	0.217
eGFR, mL/min per 1.73 m^2^	179 ± 52	159 ± 41	146 ± 35	<0.001
SUA, mg/dL	4.54 ± 1.15	4.95 ± 1.21	5.48 ± 1.28	<0.001
BUN, mg/dL	10.04 ± 3.07	10.49 ± 3.13	10.76 ± 3.43	<0.001
CRP, mg/dL	0.17 ± 0.41	0.20 ± 0.56	0.20 ± 0.53	0.208
Serum Vitamin B12, pg/mL	655.64 ± 541.57	578.89 ± 244.89	498.93 ± 201.46	<0.001
Serum folate, ng/mL	15.19 ± 5.71	12.93 ± 4.82	10.53 ± 4.24	<0.001
ALT, U/L	18.89 ± 12.56	18.75 ± 15.91	20.4]0 ± 15.64	<0.001
AST, U/L	23.70 ± 8.23	23.33 ± 9.69	24.25 ± 11.79	0.020
GGT, U/L	14.83 ± 10.05	15.53 ± 8.65	17.74 ± 12.17	<0.001

Hcy, homocysteine; BMI, body mass index; SBP, systolic blood pressure; DBP, diastolic blood pressure; FBG, fasting blood glucose; TC, total cholesterol; eGFR, estimated glomerular fltration rate; SUA, serum uric acid, BUN, blood urea nitrogen; CRP, C-reactive protein, ALT, alanine aminotransferase; AST, aspartate aminotransferase; GGT, gamma glutamyl transferase. *Mean ± SD for continuous variables: p-value was calculated by weighted linear regression model. % for categorical variables: p-value was calculated by weighted chi-square test. ^§^ BMI was calculated as the body weight in kilograms divided by the square of the height in meters.

*^†^Convert to Hcy tertiles range: <5.07 μmol/L, 5.07–6.34 μmol/L, ≥6.34 μmol/L.*

### Association of Serum Homocysteine With Serum Uric Acid

**FIGURE 1:**
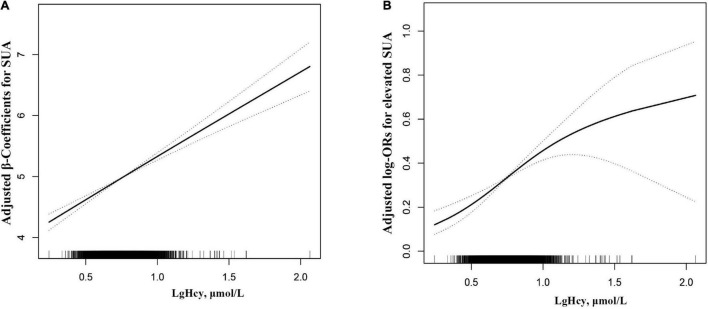
Dose-response relationship between Lg Hcy and SUA* **(A)** Lg Hcy and SUA; **(B)** Lg Hcy and elevated SUA. The solid line and dashed line represent the estimated values and their corresponding 95% confidence interval, respectively. *The adjustment factors included age, sex, BMI, SBP, DBP, non-Hispanic White, non-Hispanic Black, Mexican American, Other Hispanic, Other races, education, FBG, Triglycerides, TC, eGFR, BUN, CRP, Serum folate, Serum Vitamin B12, ALT, AST, GGT, Vitamin B12 intake, Vitamin B6 intake.

As shown in Figure 1A, there was a dose–response relationship between Hcy and SUA, and the results showed that Hcy was linearly positively correlated with SUA. The β value and 95% CIs for SUA in the three models are listed in Table 2. In model 1, the level of SUA increased by 1.65 mg/dL for each increased unit of Hcy. After further adjustment for age, sex, BMI, SBP, DBP, non-Hispanic white, non-Hispanic black, Mexican American, other Hispanic, other races, education, and other confounding factors, the results showed that the level of SUA increased by 1.62 mg/dL for each increasing unit of Hcy. In the fully adjusted model 3, the results showed that the positive correlation between Hcy and SUA remained stable. To verify whether the results are stable, we further used Hcy as a classification variable and observed its relationship with SUA. In the fully adjusted model 3, taking T1 of LgHcy as the reference, the estimated β of SUA in T2 and T3 participants increased by 0.14 mg/dL (95% CI: 0.08, 0.21) and 0.39 mg/dL (95% CI: 0.31, 0.46), respectively.

**TABLE 2:** Association of SUA with LgHcy among 5,404 12–19 year-old adolescents, NHANES 1999–2006.

	SUA, mg/dL, β (95%CI), *P*-value		
Lg Hcy, μ mol/L	Model 1	Model 2	Model 3
Per 1, μmol/L increase	1.65 (1.42, 1.88), <0.001	1.62 (1.39, 1.85), <0.001	1.43 (1.18, 1.68), <0.001
**Tertiles**			
T1 (<5.07)	0	0	0
T2 (5.07–6.34)	0.19 (0.12, 0.25), <0.001	0.19 (0.12, 0.25), <0.001	0.14 (0.08, 0.21), <0.001
T3 (≥6.34)	0.47 (0.40, 0.54), <0.001	0.46 (0.39, 0.53), <0.001	0.39 (0.31, 0.46), <0.001
*P* for trend	<0.001	<0.001	<0.001

*Model 1 was adjusted for age, sex, BMI, SBP, DBP. Model 2 was adjusted for age, sex, BMI, SBP, DBP, non-Hispanic White, non-Hispanic Black, Mexican American, Other Hispanic, Other races, education. Model 3 was adjusted for age, sex, BMI, SBP, DBP, non-Hispanic White, non-Hispanic Black, Mexican American, Other Hispanic, Other races, education, FBG, Triglycerides, TC, eGFR, BUN, CRP, Serum folate, Serum Vitamin B12, ALT, AST, GGT, Vitamin B12 intake, Vitamin B6 intake.*

Table 3 presents the relative odds of having elevated SUA levels. As shown in Figure 1B, Hcy level was positively correlated with the risk of elevated SUA in adolescents. As shown in Table 3, per 1% increment in Hcy, the risk of elevated SUA increased by 8.8 times [odds ratio (OR), 8.80, 95% CI: 4.25, 18.20] in the fully adjusted model. SUA is transformed from a continuous variable to a classified variable (tertile). The multivariable-adjusted ORs for elevated SUA when comparing T1 (<5.07) with T2 (5.07–6.34) and T3 (≥6.34) were 1.18 (95% CI: 0.97, 1.43), 1.92 (95% CI: 1.55, 2.37). *p* for trend in all the models was significant.

**TABLE 3:** Relative odds of having an elevated SUA levels among 5,404 12–19 year-old adolescents, NHANES 1999–2006.

		Elevated SUA OR (95%CI), *P*-value		
Lg Hcy, μ mol/L	Events (%)	Model 1	Model 2	Model 3
Per 1, μmol/L increase	1813 (33.55%)	13.80 (7.35, 25.91), <0.001	14.57 (7.68, 27.64), <0.001	8.80 (4.25, 18.20), <0.001
**Tertiles**				
T1 (<5.07)	362 (20.19%)	1	1	1
T2 (5.07–6.34)	556 (30.91%)	1.29 (1.07, 1.54), 0.007	1.30 (1.09, 1.57), 0.005	1.18 (0.97, 1.43), 0.090
T3 (≥6.34)	895 (49.39%)	2.20 (1.82, 2.65), <0.001	2.25 (1.86, 2.73), <0.001	1.92 (1.55, 2.37), <0.001
P for trend		<0.001	<0.001	<0.001

*Model 1 was adjusted for age, sex, BMI, SBP, DBP. Model 2 was adjusted for age, sex, BMI, SBP, DBP, non-Hispanic White, non-Hispanic Black, Mexican American, Other Hispanic, Other races, education. Model 3 was adjusted for age, sex, BMI, SBP, DBP, non-Hispanic White, non-Hispanic Black, Mexican American, Other Hispanic, Other races, education, FBG, Triglycerides, TC, eGFR, BUN, CRP, Serum folate, Serum Vitamin B12, ALT, AST, GGT, Vitamin B12 intake, Vitamin B6 intake.*

### Subgroup Analyses by Potential Effect Modifiers

**FIGURE 2:**
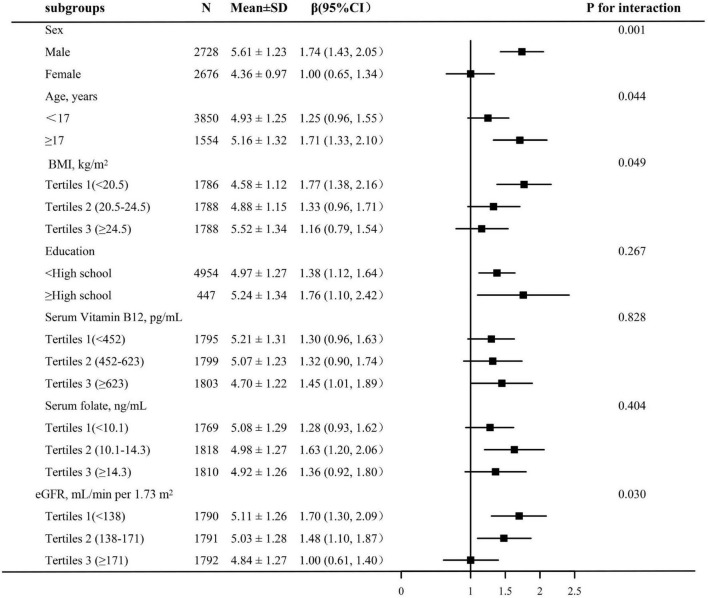
Subgroup analyses of the effect of Lg Hcy on SUA. Adjusted for age, sex, BMI, SBP, DBP, non-Hispanic White, non-Hispanic Black, Mexican American, Other Hispanic, Other races, education, FBG, Triglycerides, TC, eGFR, BUN, CRP, Serum folate, Serum Vitamin B12, ALT, AST, GGT, Vitamin B12 intake, Vitamin B6 intake, if not be stratified.

We performed a further stratified analysis to evaluate the effect of LgHcy on SUA in different subgroups. As shown in Figure 2, the relationship between LgHcy and SUA was significantly different according to sex, age, BMI, and eGFR stratification (*P* for interaction <0.05). However, the positive association between LgHcy and SUA was consistent in the following subgroups: education attainment, serum vitamin B12, and serum folate (*P* for interaction >0.05).

## Discussion

In this large, representative multi-ethnic cross-sectional study based on U.S. adolescents, it was shown that Hcy levels are positively correlated with SUA levels and elevated SUA levels. In addition, subgroup analysis showed stronger associations between Hcy and SUA among boys aged 17 years or older and teenagers with low BMI and eGFR.

There are few previous studies on the associations between Hcy and SUA, and most of them were carried out among healthy adults, patients with ASCVD, diabetes patients, and gout patients (19–23). However, the above studies have presented inconsistent conclusions with regard to the association between Hcy and SUA. Boras et al. (20) explored the relationship between Hcy levels and SUA in 52 patients with type 2 diabetes mellitus complicated with acute myocardial infarction, and the results showed that Hcy was positively correlated with SUA. Kiseljaković et al. (19) conducted a cross-sectional study of 99 patients with ASCVD and 40 healthy participants. The average ages of the ASCVD group and the healthy group were 53.62 ± 1.17 years and 57.49 ± 1.71 years, respectively. The results revealed that the Hcy level was positively correlated with SUA independently in both the ASCVD and healthy groups. A cross-sectional study conducted by Shih et al. (21)using the data of community physical examination in Taiwan Province in 2019 included 396 middle-aged and elderly patients aged 50–85 years. The results indicated that a higher Hcy level was closely related to a higher SUA level. However, a retrospective cross-sectional study conducted by Choi et al. included 91 male patients with gout and 97 healthy men. They found that Hcy levels were not related to SUA levels in gout patients (γ = −0.002, *P* = 0.988) (22). The inconsistency in the above conclusions may be due to the different confounding factors of the study population and adjustment.

It is well known that adults may be more prone to developing diabetes, gout, ASCVD, or bad habits such as smoking and drinking. Even if these are adjusted as confounding factors, the relationship between Hcy and SUA will be affected or concealed in the disease. However, in our study, we reported the relationship between Hcy levels and SUA among teenagers. This is an ideal population for evaluating the relationships between the two parameters. This study found that although the level of Hcy in adolescents is low, it has a positive correlation with SUA. Further research is needed to determine the optimal Hcy level in adolescents.

Because there is a MeT cycle in the human body, MeT can be converted into SAH, which can be converted into Hcy and adenosyl (16), and Hcy accepts a methyl group to regenerate MeT (17). Adenosine can be metabolized into uric acid (18); therefore, SUA levels in the human body can indirectly reflect the level of Hcy. The kidneys simultaneously excrete uric acid and Hcy at the same time. If the level of eGFR decreases, then Hcy will accumulate in the body, and this accumulation of Hcy will lead to kidney damage and further decrease in the eGFR level (28, 29). Loeffler et al. (14); therefore, SUA levels in the human body can indirectly reflect the level of Hcy. The kidneys simultaneously excrete uric acid and Hcy at the same time. If the level of eGFR decreases, then Hcy will accumulate in the body, and this accumulation of Hcy will lead to kidney damage and further decrease in the eGFR level (30); compared with women, the level of Hcy in men is higher because of hormones (31, 32). As an index of obesity, BMI is closely related to metabolic factors such as uric acid and Hcy. However, we found that the relationship between Hcy and uric acid was more significant in people with a lower BMI. According to a recent study, the concentration of Hcy is negatively correlated with BMI (33); therefore, the level of Hcy is lower in people with higher BMI, and in people with higher BMI, with an increase in the Hcy level, the increase in SUA level is smaller.

This study had both advantages and limitations. The advantages of this study are as follows: First, this study is the first to explore the relationship between Hcy and SUA among American teenagers with low Hcy levels. Second, we adjusted for the most potential confounding factors and effect correction factors. Finally, to reduce the contingency in data analysis and enhance the robustness of the results, we treated independent variables as continuous and classified variables. However, the limitations are as follows: First, this is an observational cross-sectional study; hence, we cannot infer a causal relationship between the two parameters. Therefore, further prospective follow-up studies are needed to confirm the conclusions of this study. Second, this study collected Hcy data only once at the baseline, and multiple tests may make the results more accurate. Third, because of the problem of data collection, diets that affect the SUA level, for example, alcohol, meat, coffee, fruits and vegetables, and dairy products, have not been adjusted for. However, we adjusted for many confounding factors in this study. Finally, this study was conducted among American teenagers, and whether its conclusions can be extended to other groups remains to be discussed.

## Conclusion

In conclusion, there is a positive correlation between Hcy levels and SUA levels among U.S. teenagers, and this effect is more significant among boys aged ≥17 years and among people with lower BMI and eGFR.

## Data Availability Statement

Publicly available datasets were analyzed in this study. This data can be found here: https://www.cdc.gov/nchs/nhanes/index.htm.

## Ethics Statement

The studies involving human participants were reviewed and approved by the Research Ethics Review Board of the National Center for Health Statistics. Written informed consent to participate in this study was provided by the participants’ legal guardian/next of kin.

## Author Contributions

YS participated in literature search, study design, data collection, data analysis, data interpretation, and wrote the manuscript. ZW, JW, and ZC conceived the study, and participated in its design, coordination, data collection, and analysis. PL participated in study design and provided the critical revision. All authors read and approved the final manuscript.

## Conflict of Interest

The authors declare that the research was conducted in the absence of any commercial or financial relationships that could be construed as a potential conflict of interest.

## Publisher’s Note

All claims expressed in this article are solely those of the authors and do not necessarily represent those of their affiliated organizations, or those of the publisher, the editors and the reviewers. Any product that may be evaluated in this article, or claim that may be made by its manufacturer, is not guaranteed or endorsed by the publisher.

## Footnotes

1
https://wwwn.cdc.gov/nchs/nhanes/tutorials/default.aspx

2
http://www.r-project.org

3
www.empowerstats.com
