# Supplementary figures and images for: Serum Homocysteine Level Is Positively Correlated With Serum Uric Acid Level in U.S. Adolescents: A Cross Sectional Study
Source: Front Nutr. 2022 Mar 29;9:818836. doi: 10.3389/fnut.2022.818836 (PMC9001925; doi:10.3389/fnut.2022.818836)

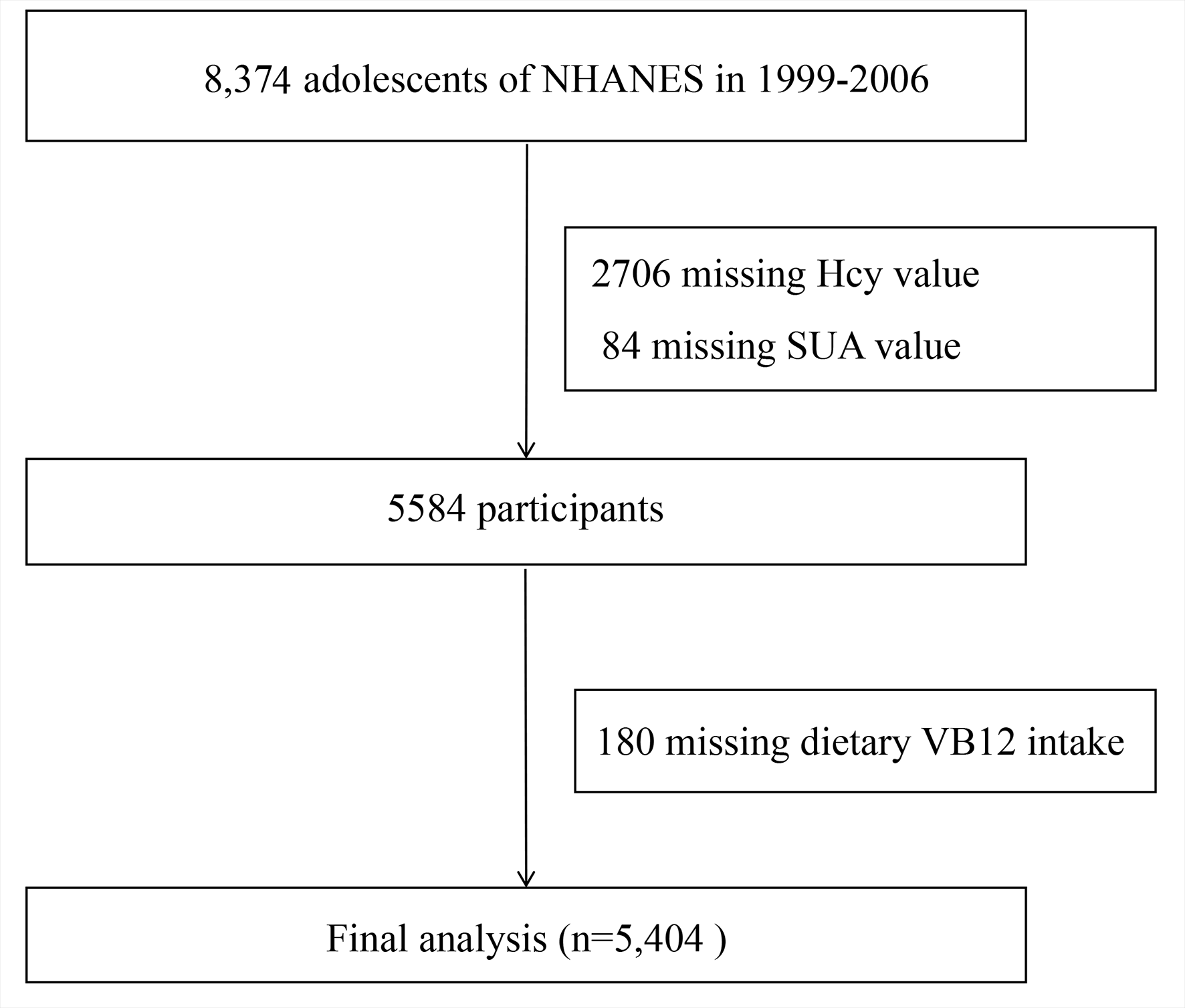

Supplement: Supplementary file 1 [file Image_1.TIF]
